# Semi-supervised learning for colposcopic image classification using generative adversarial networks

**DOI:** 10.3389/fmed.2026.1828859

**Published:** 2026-07-23

**Authors:** Feng Liang, Yun Feng, Jin Ding, Chuanyi Zhang, Junyao Sun, Shue Wang, Cheng Peng, Gangming Zhao, Liping Li

**Affiliations:** 1 Key Laboratory of Intelligent Computing and Signal Processing, MOE, Anhui University, Hefei, China; 2 Department of Obstetrics and Gynecology, Zhongda Hospital, School of Medicine, Southeast University, Nanjing, Jiangsu, China; 3 The People's Hospital of Fanchang District, Wuhu, Anhui, China; 4 The Traditional Chinese Medicine Hospital of Taihe, Taihe, Anhui, China; 5 Department of Obstetrics and Gynecology, The First Affiliated Hospital of USTC, Division of Life Sciences and Medicine, University of Science and Technology of China, Hefei, Anhui, China

**Keywords:** classification of cervical lesions, colposcopic image, deep learning, generative adversarial networks, semi-supervised learning

## Abstract

**Introduction:**

Traditional colposcopy screening faces several challenges, including heavy reliance on physician expertise, relatively high misdiagnosis rates, and uneven distribution of primary healthcare resources. To address these limitations, this study aimed to develop an artificial intelligence-assisted framework for colposcopic image generation and interpretation.

**Methods:**

We proposed a Feature-pyramid and Squeeze-excitation Generative Adversarial Network (FSGAN) to generate realistic colposcopic images and a region-focused recognition model named CenSwin Transformer for image classification. FSGAN-generated images were incorporated into model training, and a dynamic pseudo-label semi-supervised learning strategy was applied to improve recognition performance. The proposed model was evaluated through 12 repeated experiments and further validated on an additional independent dataset.

**Results:**

Across 12 repeated experiments, CenSwin Transformer achieved an accuracy of 90.39 ± 0.67%, recall of 86.10 ± 4.33%, and F1-score of 0.8645 ± 0.0111. Compared with Swin Transformer, CenSwin Transformer showed statistically significant improvements in accuracy and F1-score. On the independent validation dataset, the model achieved an accuracy of 85.26% and recall of 71.11%.

**Discussion:**

The proposed FSGAN-assisted semi-supervised learning framework and CenSwin Transformer model improved colposcopic image recognition performance, particularly in accuracy and F1-score. These findings suggest that the model may serve as an auxiliary second-opinion tool for colposcopic image interpretation, especially in primary healthcare settings or regions with limited access to experienced colposcopists.

## 1 Introduction

Cervical cancer ranks among the most common cancers in women worldwide, as per global cancer survey statistics, with its incidence and mortality rate positioned fourth (1). Based on the severity of cervical precancerous lesions, the World Health Organization (WHO) categorizes CIN into three grades: CINI, CINII, and CINIII. CINI, classified as Low-grade Squamous Intraepithelial Lesion (LSIL), typically undergoes pharmacological treatment followed by observation, while CINII, CINIII, and cancer, categorized as High-grade Squamous Intraepithelial Lesion (HSIL), necessitate further diagnosis and treatment. Currently, there are three cervical lesion screening methods: Human Papillomavirus (HPV) testing, Pap smear, and colposcopy (2, 3). The first two methods are relatively complicated and expensive. Colposcopy, due to its simplicity, low cost, and rapid process, has become an essential auxiliary tool for cervical cancer screening in developing countries and low-income nations (4).

Expert physicians can determine the presence of precancerous lesions through a combined visual assessment using acetic acid, Lugol's iodine solution, and colposcopy (5, 6). Thus, the accuracy of colposcopy results is closely linked to the subjective experience of the examiner. However, especially in middle and low-income countries, there are not enough experienced colposcopy experts to meet the screening demands of numerous patients (7).

## 2 Related work

Proposals have been made to utilize classical networks such as AlexNet, ResNet, and DenseNet for feature extraction and classification of lesions in colposcopy acetic acid images (8–11), aiming to assist clinicians in triage and improve the diagnostic efficiency of colposcopy physicians.

The works of Yuan (9), Alyafeai (12), and Li (13) have all utilized ResNet as the backbone network for feature extraction. Yuan et al. extracted features from acetic acid and iodine images using ResNet. Alyafeai et al. integrated the structure of ResNet with GoogleNet and used transfer learning. Li et al. employed ResNet as the backbone network to extract features from acetic acid images across multiple time slots.

Buiu et al. (14) used MobileNetV2 as the backbone network for feature extraction, separately extracting features from acetic acid solution images. They then superimposed the feature images, achieving an accuracy of 83.33%.

Fan (15) and Guo (16) both used EfficientNet for feature extraction. Fa extracted features from acetic acid and iodine images, designed a network utilizing the SE (Squeeze-and-Excitation) attention mechanism and ASPP (Atrous Spatial Pyramid Pooling) to capture multi-scale information, and performed one-hot encoding on textual information. Guo et al. incorporated CBAM (Convolutional Block Attention Module) and CA (Coordinate Attention) attention mechanisms within EfficientNet, ultimately achieving an accuracy of 90.0%. Additionally, some scholars have designed their own unique networks using convolutional neural layers to extract features for classification (17–19), also achieving relatively good results.

However, existing methods face critical challenges. The performance of deep networks highly depends on large-scale, high-quality annotated data, while the construction of colposcopic image datasets encounters multiple practical bottlenecks, including sample scarcity and class imbalance. The annotation of cervical images is costly, relying on experienced gynecologists to interpret subtle lesions such as acetowhite epithelium and mosaic vessels, which is time-consuming and labor-intensive, with low inter-observer consistency. Traditional data augmentation methods like rotation and flipping can partially alleviate over fitting, but they are limited to geometric or photo metric transformations and cannot generate new lesion patterns similar to real colposcopic images.

To address the scarcity of medical image data, Generative Adversarial Networks (GANs) (20) have shown unique advantages. Since their introduction in 2014, GANs and their variants have been applied to non-medical images and later extended to medical imaging, achieving breakthroughs in medical image synthesis. Maria J's method (21) used deep convolutional GANs (DCGANs) (22) to synthesize lung cancer nodules, which were indistinguishable from real nodules in visual tests by radiologists. DCGANs have also been used to synthesize realistic prostate lesion patches (23) and retinal images (24). Christoph Baur used progressive growing GANs (PGGANs) (25) to synthesize highly realistic high-resolution images of skin lesions (26), which dermatologists could not reliably distinguish from real samples.

More importantly, synthetic data has been proven to effectively improve classification performance. Maayan Frid-Adar et al. validated the superiority of GAN-augmented data over traditional augmentation methods in brain tumor classification tasks (27). Qi achieved mutual conversion (28) between tumor and normal images using CycleGAN (29), significantly improving classifier robustness. These successful cases provide important insights for colposcopic image data augmentation by generating synthetic images with pathological features. To address the limitations of subjectivity in traditional expert-dependent diagnosis and the scarcity of annotated data in existing studies, this paper constructs two models: a Feature-pyramid and Squeeze-excitation Generative Adversarial Network (FSGAN) to generate realistic colposcopic images, and a classification network named CenSwin Transformer to perform classification of colposcopic images (diagnosis). FSGAN integrates Feature Pyramid Network (FPN) (30) connections and a Cross-Layer Channel Excitation Module (SLE) (31) to enhance multi-scale feature fusion and generate colposcopic images with pathological features close to real images. This model is capable of synthesizing realistic colposcopic images with pathological feature diversity, overcoming the inherent limitations of sample scarcity and class imbalance in real datasets, thereby addressing model overfitting and collapse issues caused by insufficient training data.

Through these innovations, the proposed framework achieved satisfactory cervical precancerous lesion recognition and showed improved performance over several baseline models, as well as potential auxiliary value in image-only comparison with gynecologists.

## 3 Model

### 3.1 The proposed FSGAN

To address the problems of over fitting and collapse of traditional GANs as stated in the previous section, this paper designs an FSGAN based on a cross-layer channel excitation module and feature pyramid connections for multi-scale fusion to enhance gradient flow in the generator.

**Figure 1:**
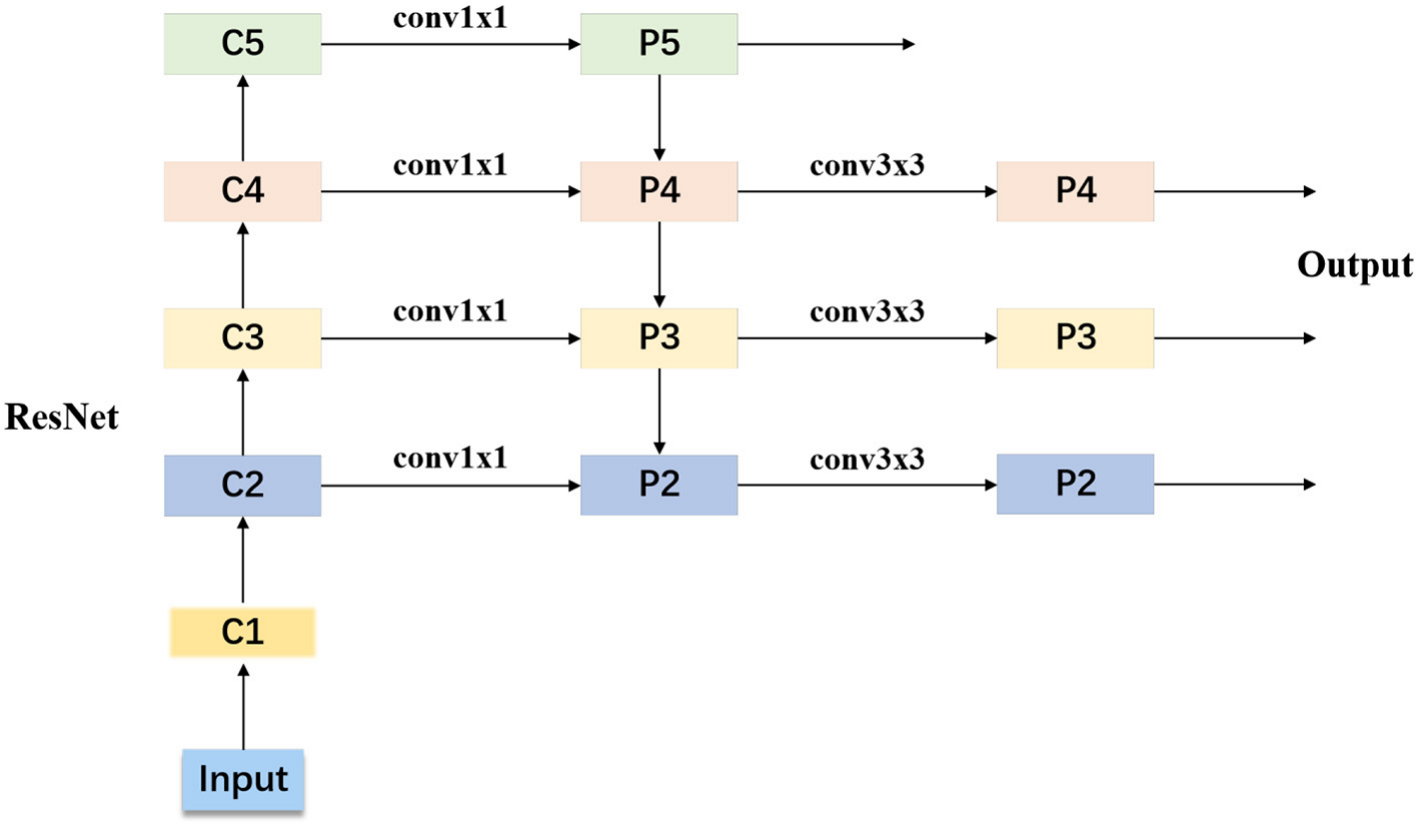
Schematic diagram of the FPN structure. This figure demonstrates the complete process of FPN's bottom-up backbone feature extraction, top-down semantic backpropagation, and cross-layer horizontal connection.

The Feature Pyramid Network (FPN) realizes the extraction and fusion of multi-scale features mainly through bottom-up feature extraction, top-down feature fusion, horizontal connection, and the construction of feature pyramids, and the combination of all these parts. FPN constructs a pyramid feature map containing multiple scales from the bottom to the top, and each feature map integrates different levels of feature information as shown in Figure 1. Among them, the bottom-up feature extraction gradually decreases the resolution of the feature map, but gradually enriches the semantic information. The top-down feature fusion matches the high-level feature map with the low-level feature map. The horizontal connection combines the high-resolution information of the low-level feature map with the rich semantic information of the high- level feature map to form a new feature map.

**Figure 2:**
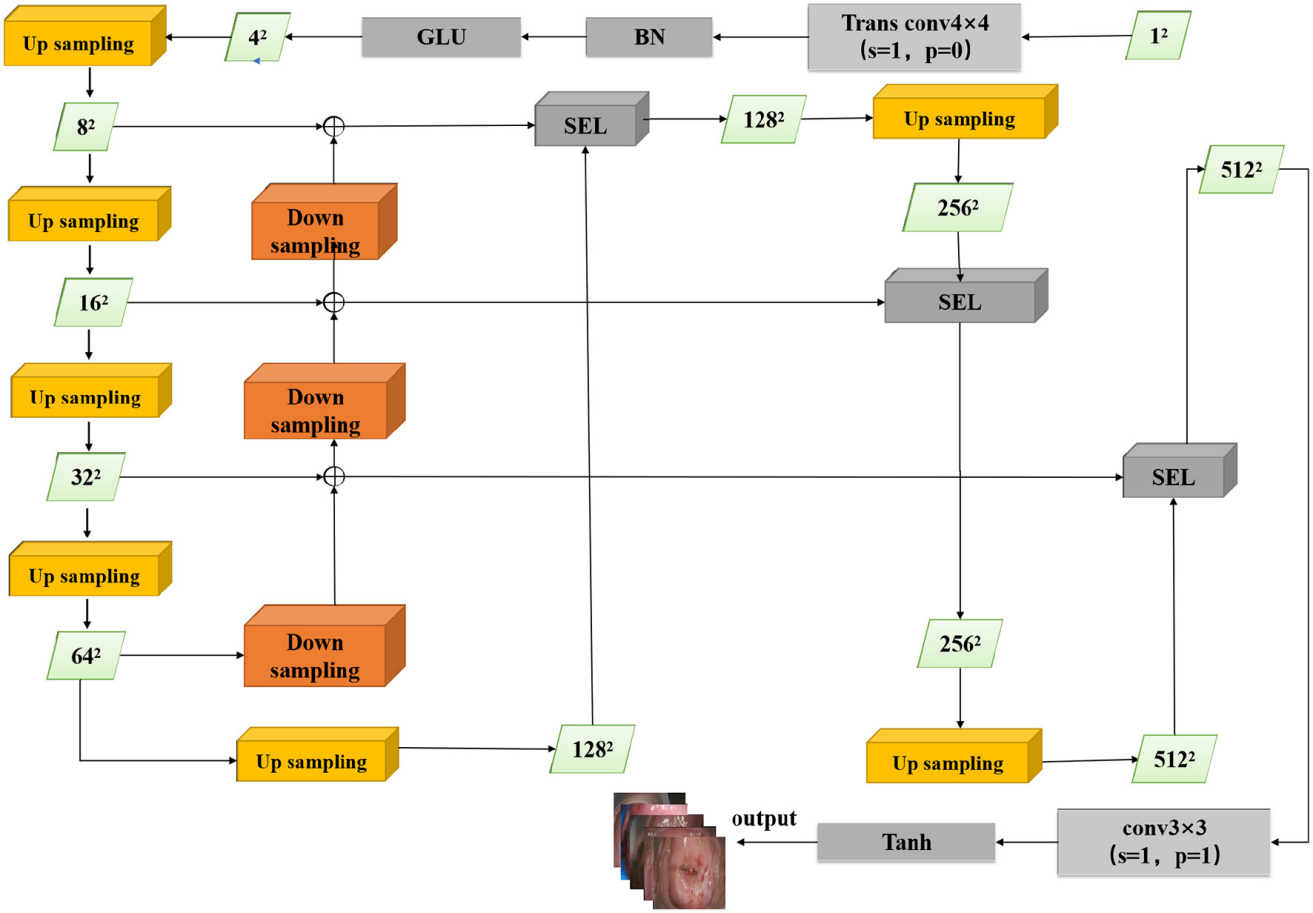
Schematic diagram of the FSGAN generator structure. After the depth and shallowness features are re-corrected in the SLE module, a 512*512 color image is output through two-stage up-sampling. This design significantly enhances the gradient flow and detail restoration capabilities.

The FSGAN generation network first transposes the convolution operation of the input one-dimensional noise vector, and then obtains the feature map of size 4*4 through batch normalization and GLU activation function. The output image is obtained by sequentially processing the feature maps through up-sampling and down-sampling modules. The entire network structure is shown in Figure 2.

**Figure 3:**
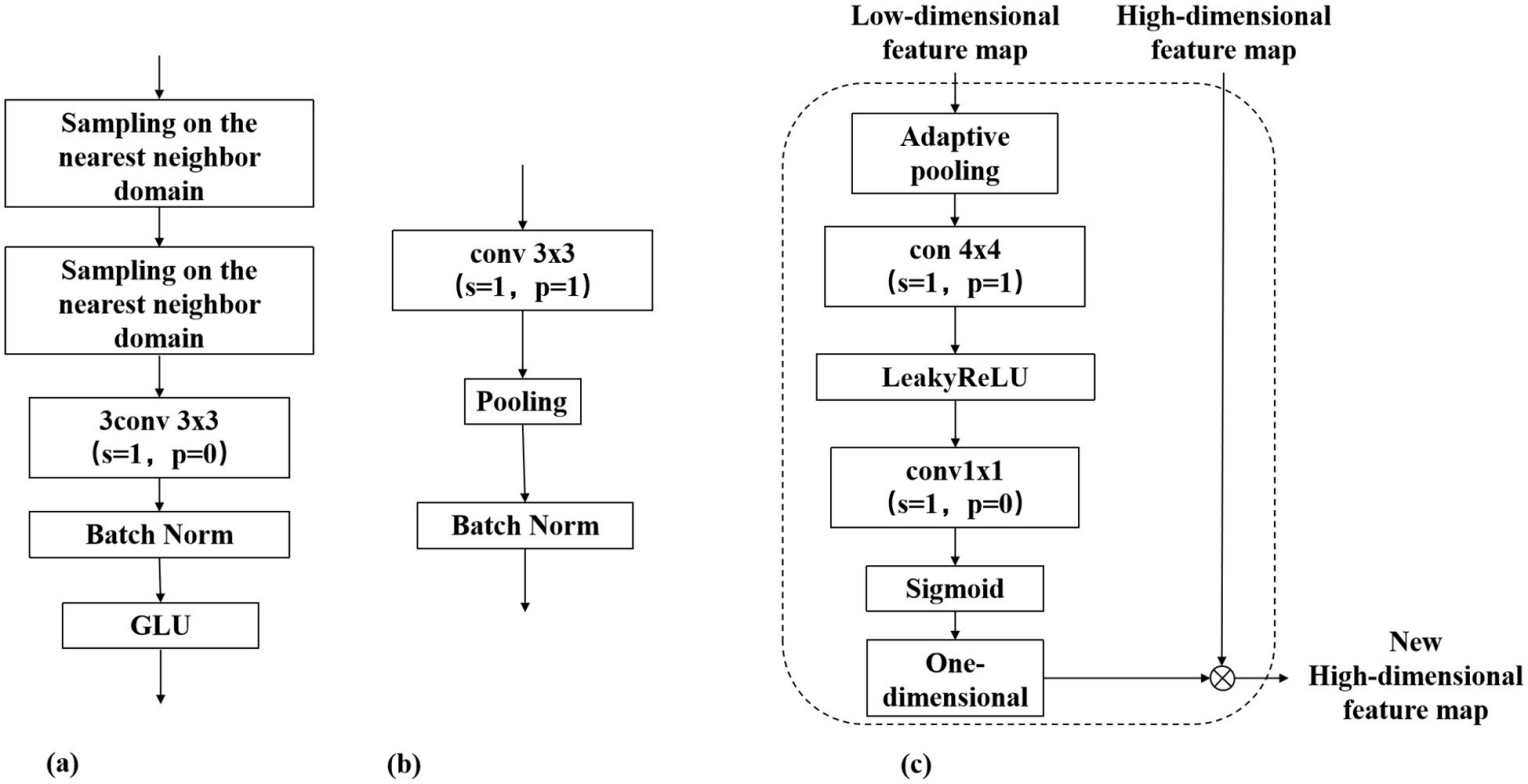
Schematic diagram of the modules in the FSGAN generator. **(a)** Up-sampling module: Two nearest neighbor interpolations followed by 3*3 convolution, BatchNorm, and GLU, effectively enlarging the spatial size and suppressing artifacts; **(b)** Down-sampling module: 3*3 convolution and pooling are performed in parallel to reduce resolution, and Stride(s) is 1, Padding(p) is 1, while BatchNorm maintains distribution stability; **(c)** Cross-layer channel excitation module: First, adaptively pool the low-resolution features, then generate channel attention through 4*4 convolution -LeakyReLU-1*1 convolution. After Sigmoid standardization, it is multiplied channel by channel with the high-resolution features to achieve semantic-texture alignment. **(a)** Up-sampling. **(b)** Down-sampling. **(c)** Cross-layer incentive.

The up-sampling module processes input feature maps through a two stage nearest-neighbor interpolation procedure followed by convolutional operations. This spatial resolution enhancement pathway incorporates batch normalization for stable gradient propagation, culminating in gated linear unit (GLU) activation for feature selection, as illustrated in Figure 3a. The counterpart down-sampling module implements progressive feature abstraction through convolutional filtering coupled with pooling operations. These processed features undergo batch normalization for distribution stabilization before final output generation, with the complete data reduction workflow depicted in Figure 3b.

The cross-layer excitation module processes multi-scale feature representations through parallel pathways, accepting both low-dimensional and high-dimensional feature maps as concurrent inputs to enhance local detail generation fidelity. The computational workflow initiates with adaptive pooling applied to the low-resolution feature maps, followed by 4*4 convolutional filtering with unitary stride and zero padding specifications.

This processed feature stream undergoes nonlinear transformation via LeakyReLU activation before dimensional alignment through point wise (1*1) convolution, maintaining identical channel dimensionality with the accompanying high-resolution feature maps. The module culminates with sigmoid-activated normalization, producing 1*1 resolution attention weights that undergo channel-wise multiplication with the original high-scale features. The output is normalized using a sigmoid function and multiplied channel-wise with the high-scale feature map (Figure 3c).

**Figure 4:**
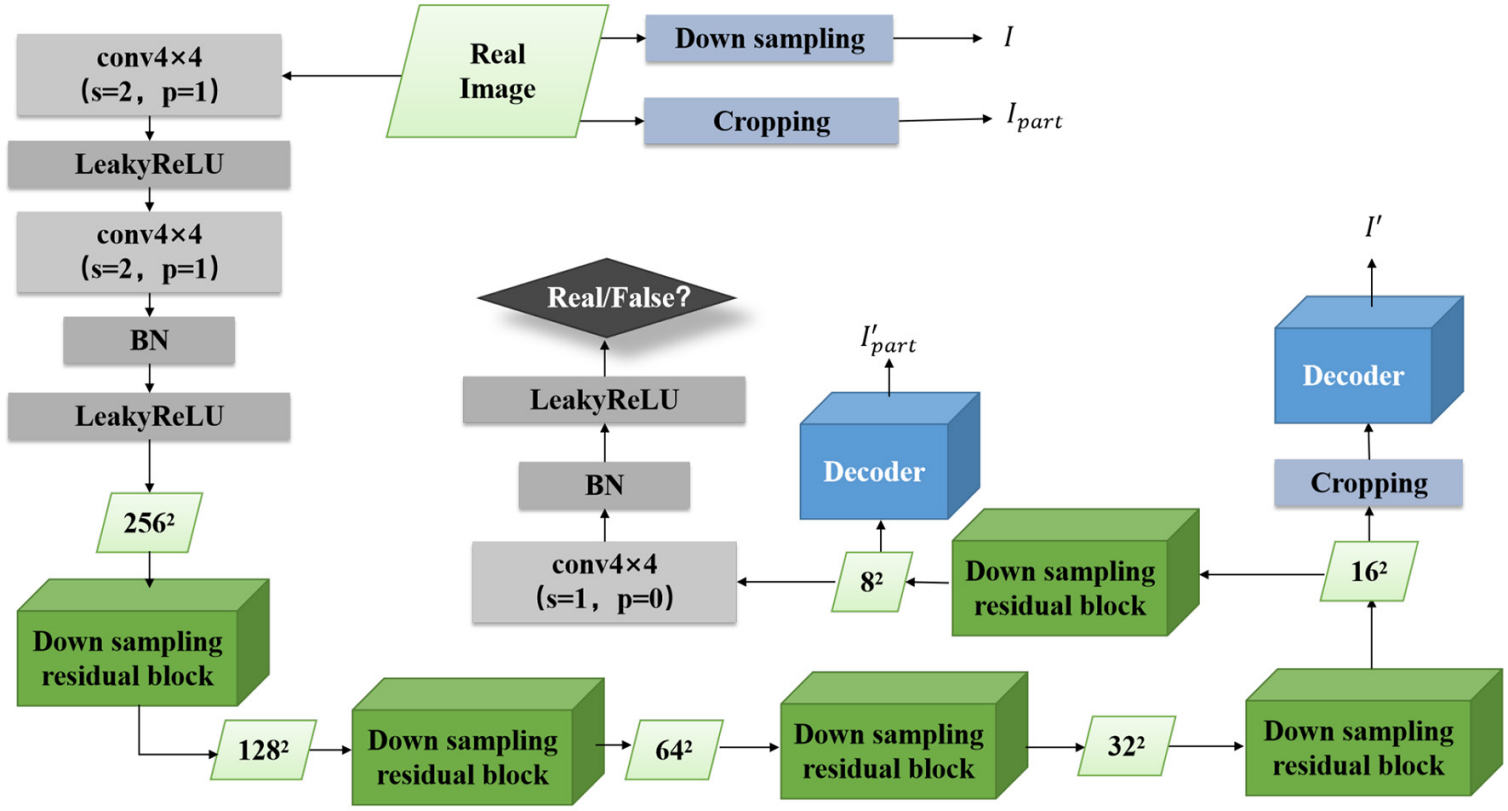
Structure diagram of the FSGAN discriminator. The discriminator consists of an encoder-discriminator dual branch and a decode-reconstruction branch. At the encoding end, multi-level residual down-sampling is carried out to 8*8, which not only outputs the authenticity discrimination logits but also sends features to the symmetric decoder, using L1 reconstruction loss to supervise texture integrity. The discriminator down-samples the real image to obtain the feature map I, crops it to get *I*_*part*_, and the decoder transforms the randomly cropped feature map into *I*′. The randomly cropped 16*16 feature map is processed by the decoder and up-sampled to 128*128's $I_{part}^{\prime }$. The 8*8 feature map is decoded and upsampled to 128*128's *I*′. This combined training of “discrimination + self-supervision” can significantly enhance the recognition of real cervical textures and the feedback strength of the generator.

**Figure 5:**
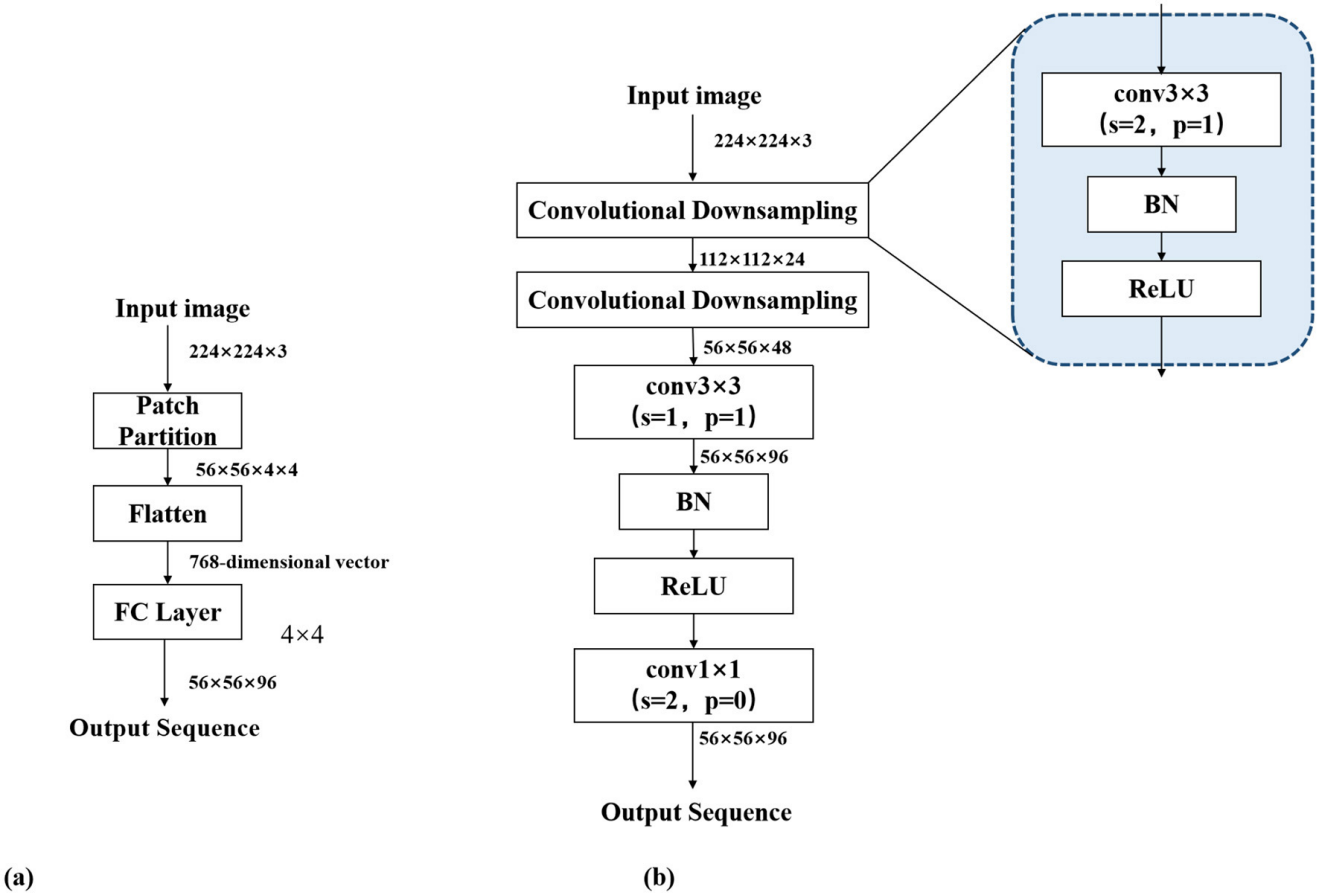
Down-sampling residual block and decoder in the FSGAN discriminator. Residual blocks are connected through a 2*2 mean pooling –1*1 convolution shortcut to avoid semantic loss; The main branch extracts discriminative features by stacking 4*4 and 3*3 convolution. The decoder adopts a four-level cascade of “nearest neighbor interpolation × 2 + 3*3 convolution + GLU” to reconstruct the randomly cropped 16*16/8*8 features to 128*128, which can strongly constrain D to retain local details such as lesion texture and vascular direction. And s means stride, *p* means padding. **(a)** Down-sampling residual block. **(b)** Decoder.

The discriminator module incorporates a self-supervised learning paradigm augmented with dedicated feature decoding capabilities for image reconstruction. When inputting real images, the feature encoder and discriminator are optimized through reconstruction loss. The structure is shown in Figure 4. The specific structure of the down-sampling residual block and the decoder is shown in Figure 5.

To enhance the discriminator's ability to learn real image distributions and better capture texture details, a reconstruction loss based on L1-norm is introduced. This loss function compares the difference between decoder reconstructed images and original down-sampled images, forcing the discriminator to preserve more detailed information during feature extraction. In implementation, the network uses feature representations from real samples for image reconstruction, optimizing parameters by minimizing reconstruction errors. The specific reconstruction loss is computed as the L1-norm between reconstructed images and down-sampled originals, as shown in Equation 1:


$$L_{\text{recons}}=E_{f\sim D_{\text{encod}}(x),x\sim I_{\text{real}}}\left[\left\|\varphi (f)-\delta (x)\right\|\right]$$ (1)

where *f* is the intermediate feature map from D (Discriminator), φ represents the processing of *f* and the decoder, and δ represents the processing of the real image sample *x*.

This reconstruction loss is only applied to real images, ensuring that the discriminator D extracts more comprehensive representations, including overall composition and detailed textures. The hinge version of adversarial loss is used as the discriminator loss shown in Equation 2:


$$\begin{array}{l} L_{D}=-E_{x\sim I_{\text{real}}}\left[\min\left(0,-1+D\left(x\right)\right)\right]\\ \;-\;-\;E_{\hat{x}\sim G\left(z\right)}\left[\min\left(0,-1-D\left(\hat{x}\right)\right)\right]+L_{\text{recons}} \end{array}$$ (2)

where the third term is the reconstruction loss as Equation 1. D(*x*) is the output of the discriminator. For real images, D(*x*) is maximized to be as large as possible (≥1), while for fake images, D(*x*) is minimized to be as small as possible ( ≤ −1).

### 3.2 CenSwin Transformer

**Figure 6:**
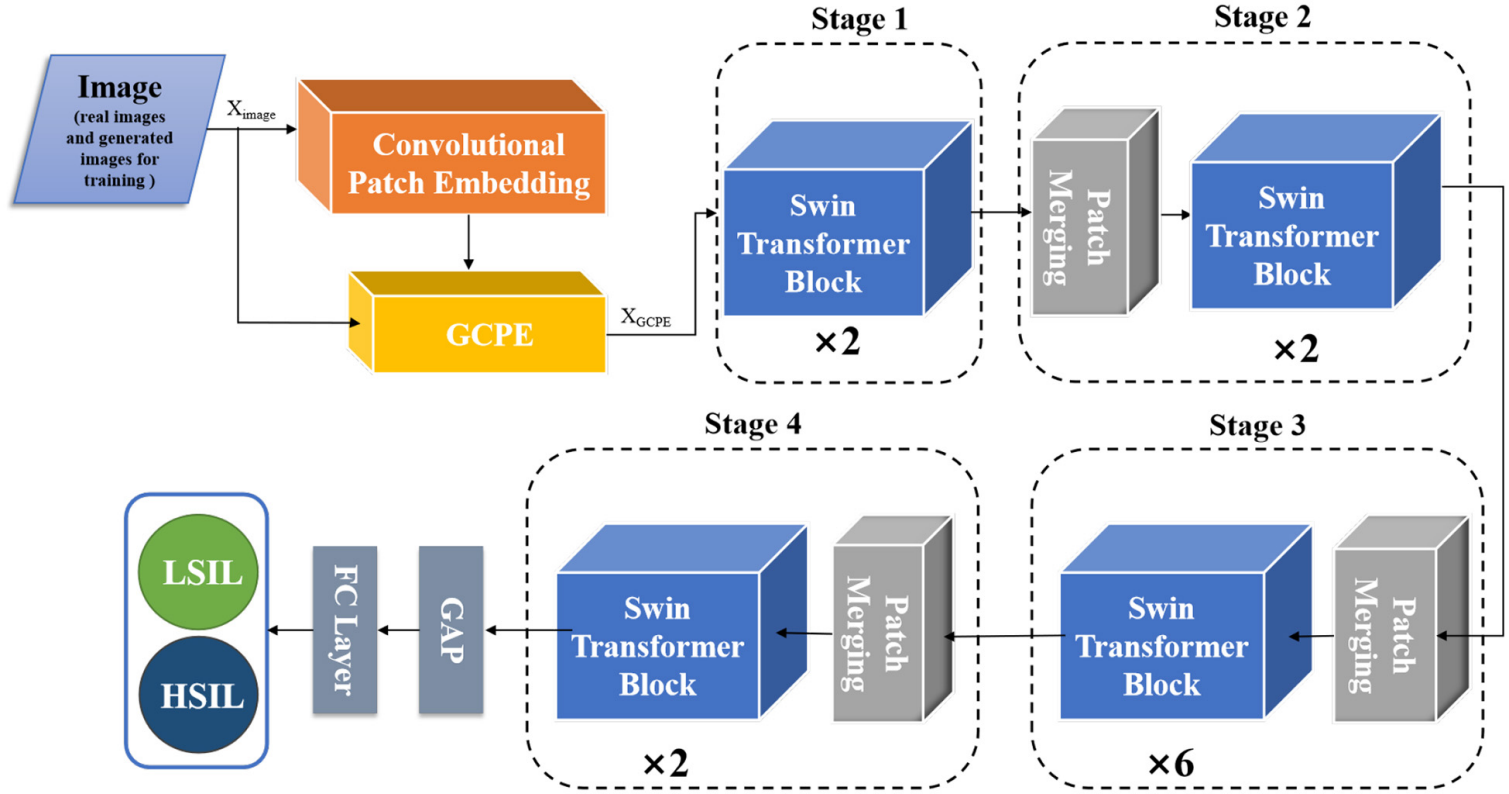
Structure diagram of the CenSwin Transformer network. The figure presents a 4-level feature pyramid based on convolutional patch embedding. The Central Position global encoding (GCPE) is weighted by standardized Euclidean distance and fused into the 96-dimensional embedding through lightweight MLP, thereby dynamically enhancing the saliency of the cervical transformation area (the center of the image). The subsequent four Swin Transformer stages complete local-global attention modeling at resolutions of 56 → 28 → 14 → 7, and the end-global average pooling FC output binary classification probability.

The proposed CenSwin Transformer is designed to enhance lesion-related feature representation in the central cervical transformation zone while suppressing peripheral noise. As shown in Figure 6, the network consists of three major components: convolutional patch embedding, global central position encoding (GCPE), and the Swin Transformer backbone. First, the input colposcopic image is resized to 224 × 224 and divided into non-overlapping patches. Different from the original linear patch projection used in ViT, a convolutional patch embedding layer is adopted to preserve local spatial continuity and improve robustness to complex colposcopic backgrounds. Second, GCPE generates a center-aware positional encoding according to the normalized Euclidean distance between each pixel and the image center. This encoding assigns relatively higher spatial weights to the central transformation zone and lower weights to peripheral regions that may contain speculum artifacts, vaginal wall interference, or blood contamination. Finally, the fused features are fed into the hierarchical Swin Transformer backbone, where window-based self-attention progressively captures local and global contextual information. The output features are aggregated by global average pooling and passed through fully connected layers for binary classification. Precancerous and cancerous lesions typically occur in the cervical transformation zone, located near the external cervical os. This zone is the transitional area between the squamous epithelium (flat, multilayered cells) of the cervical surface and the columnar epithelium (single-layered columnar cells secreting mucus) inside the cervical canal, corresponding to the central field of view in colposcopic images. However, clinically captured colposcopic images often contain interference factors such as instrument obstruction, vaginal walls outside the cervix, and blood contamination in non-diagnostic regions. To address this issue, we design a recognition model, CenSwin Transformer, with the ability to focus on the central region (transformation zone) of cervical images. With this ability, the designed CenSwin transformer can enhance feature extraction for high-risk areas while suppress noise from peripheral interference, improving the model's sensitivity to CIN and early cancerous features.

The CenSwin Transformer improves upon Swin Transformer by mimicking the human peripheral vision system. It simplifies PerViT's (32) complex ring attention division and dynamic projection mechanism, achieving a more efficient bionic design with a lightweight Multi-Layer Perceptron (MLP).

The proposed processing for the aforementioned focus on the central region is called a Global Central Position Encoding (GCPE) scheme that mimics the foveal high-resolution perception of central vision. The methodology begins by computing normalized Euclidean distance for each pixel relative to the image center, as shown in Equation 3:


$$\begin{array}{l} d_{\text{global}}=\frac{\sqrt{{\left(x-W/2\right)}^{2}+{\left(y-H/2\right)}^{2}}}{\sqrt{{\left(W/2\right)}^{2}+{\left(H/2\right)}^{2}}} \end{array}$$ (3)

where pixel coordinates are (*x, y*) of original image, and the image center coordinates are (*W/2, H/2*) (*H* is the height of the image, and *W* is the width).

This normalization process in Equation 3 constrains all distance values to a standardized range, enabling the model to systematically quantify spatial relationships between every pixel and the image center.

Then, we design a lightweight MLP to map the one-dimensional normalized distance to a high-dimensional feature space as Equation 4:


$$\begin{array}{l} P_{i,j}=\text{MLP}\left(d_{global}\right)\in R^{C} \end{array}$$ (4)

The proposed structure further replaces the linear patch projection in ViT (33) with a convolutional patch embedding layer, preserving spatial relationships between pixels and enhancing robustness to complex backgrounds. Fuse the position encoding with the output of convolutional patch embedding by pixel-wise addition (Equation 5), ensuring the feature dimension remains unchanged. This fusion method directly modifies the numerical distribution of input features, significantly enhancing the feature values of pixels near the image center while relatively weakening those at the edges, implicitly guiding the model to focus on the central region.


$$\begin{array}{l} X_{GCPE}=\text{Conv-Patch-Embedding}\left(X_{\text{image}}\right)+\left(P_{i,j}\right) \end{array}$$ (5)

After the previous processing, edge pixels naturally attenuate in feature weight during attention calculation as their encoding values approach zero. Compared to PerViT, the parameter count is reduced by over 90%, with almost no additional computational cost, and no reliance on multi-stage attention map supervision, significantly lowering training costs.

The overall architecture of the proposed CenSwin Transformer is shown in Figure 6. It integrates convolutional patch embedding, global central position encoding, and a hierarchical Swin Transformer backbone to enhance central lesion-related features while reducing the influence of peripheral interference. The input images first undergo preprocessing to the standard 224*224 resolution before entering the network. The GCPE module processes the input images as previously described to generate 96-dimensional positional encoding vectors. The results are added pixel-wise to the convolutional patch embedding as shown in Equation 5. The processed feature maps are fed into the Swin Transformer backbone network, undergoing sequential processing through four distinct stages of window-based attention mechanisms and down-sampling operations.

The network culminates with a Global Average Pooling (GAP) operation followed by Full Connection (FC) layers that transform the learned representations into class probability distributions.

### 3.3 Self-training for CenSwin Transformer

With the generating realistic colposcopy images using the FSGAN designed in this study, the proposed CenSwin Transformer is used as the backbone network for the recognition of the degree of cervical precancerous lesions, using a semi-supervised training strategy that fused progressive confidence screening and dynamic pseudo-label updating.

Self-training is a key step in semi-supervised learning, demonstrating significant advantages in scenarios where some data is labeled and the rest is unlabeled. It is closely related to pseudo-labeling technology: pseudo-labeling specifically refers to the process of assigning labels to unlabeled data using model predictions, while self-training is a complete learning framework based on pseudo-label generation, combined with data screening and iterative training.

The pseudo-labeling technique effectively promotes the realization of the goal of entropy minimization (34) by forcing the model to make low-entropy (high-confidence) predictions on unlabeled data, thereby promoting the shift of the decision-making boundary to low-density areas.

The self-training loss function needs to consider the contribution of both labeled data and pseudo-labeled data. Its mathematical expression can be defined as Equation 6:


$$\begin{array}{l} L=\frac{1}{n}\overset{n}{\underset{m=1}{\sum }}\overset{C}{\underset{i=1}{\sum }}L\left(y_{i}^{m},f_{i}^{m}\right)+\alpha \left(t\right)\frac{1}{n^{\prime }}\overset{n^{\prime }}{\underset{m=1}{\sum }}\overset{C}{\underset{i=1}{\sum }}L\left({ỹ}_{i}^{m},{\tilde{f}}_{i}^{m}\right) \end{array}$$ (6)

the first term in the summation is the supervision loss, and the second is the pseudo-label consistency loss, which is only used after the pseudo-label is injected. In Equation 6, nandn' are the quantities of labeled and unlabeled data, respectively, $f_{i}^{m}$ is the predicted output of the model for the *m*−*th* sample in the *i*−*th* class, ${\tilde{f}}_{i}^{m}$ is the predictive output for the pseudo-labels, and $y_{i}^{m}$ and ${ỹ}_{i}^{m}$ are the one-hot encoding of the true label and pseudo-label, respectively.

Firstly, the colposcopy images with real labels in the original dataset are used for preliminary supervised training of CenSwin Transformer, so that the model can establish the basic discrimination ability of lesion features. When the model converges to a steady state, the pseudo-label generation stage is started: the images generated by FSGAN are input into the model, and reliable samples are screened through the progressive confidence threshold control mechanism. Setting a strict threshold at the initial stage and keeping only the predictions with a high degree of confidence as pseudo-labels can effectively control the risk of noise injection in the early stages. With the progress of the training process, the model's ability to capture lesion features is gradually enhanced, the confidence threshold is dynamically lowered, and the pseudo-label sample pool is gradually expanded, so that the model can gradually learn more challenging boundary sample features.

In the hybrid training stage, the original labeled data and the newly generated pseudo-labeled data participate in the loss calculation in a weighted form. By introducing the periodic pseudo-label update strategy, the prediction results of the unlabeled data are re-evaluated at every fixed training round, and the updated pseudo-labels are generated by using the latest parameters of the model. This dynamic refresh mechanism forms a positive feedback loop of model optimization, label update, and re-optimization.

## 4 Evaluation criteria

In this paper, our evaluation criteria include accuracy, recall, F1-score, precision and specificity to assess the model's performance. Accuracy is the most commonly used metric in classification models, describing the proportion of samples correctly predicted by the classifier. Recall, also known as sensitivity, refers to the proportion of true positive samples successfully predicted as positive by the classifier among all actual positive instances. The F1-score is the harmonic mean of precision and recall, which comprehensively considers both precision and recall to evaluate the overall performance of the model. It ranges from 0 to 1, with values closer to 1 indicating better model performance. Precision measures the proportion of true positive samples among those predicted as positive by the model, specifically indicating the proportion of correctly identified high-risk patients among all samples predicted as high risk by the model. In other words, it represents how many of the samples predicted as high risk are actually high risk. Specificity measures the proportion of true negative samples among those predicted as negative or low risk (negative), indicating the proportion of correctly identified negative samples among all samples predicted as negative by the model. In essence, it represents how many of the samples predicted as negative are truly negative.

In addition, receiver operating characteristic area under the curve (ROC-AUC) and precision-recall area under the curve (PR-AUC) were further used for representative models and generated-image settings to evaluate threshold-independent discriminative ability. ROC-AUC reflects the trade-off between sensitivity and false-positive rate across different thresholds, while PR-AUC reflects the trade-off between precision and recall and is particularly informative under class imbalance.

### 4.1 Statistical analysis

To provide a more reliable comparison, each classification network was evaluated over 12 repeated experiments under the same experimental protocol. The results are reported as mean ± standard deviation. The 95% confidence intervals were calculated using the *t*-distribution. Since the repeated experimental results were paired across different methods and experimental settings, the two-sided Wilcoxon signed-rank test was used to evaluate whether the performance differences were statistically significant. A *p*-value < 0.05 was considered statistically significant.

## 5 Experimental process and results

### 5.1 Environment

All experiments were conducted on an NVIDIA Quadro RTX 8000 GPU with CUDA v11.0. All deep learning models were trained using Python v3.8, PyTorch v1.12.0, torchvision v0.13.0, Matplotlib v3.7.2, and NumPy v1.24.3. All experiments were performed under the same computational environment.

### 5.2 Generate realistic image with FSGAN

#### 5.2.1 Dataset for FSGAN

The cervical dataset used in this experiment was provided by Yijishan Hospital of Wannan Medical College, the First Affiliated Hospital of Anhui Medical University, Taihe County Hospital of Traditional Chinese Medicine, and Xuancheng People's Hospital. A dilute acetic acid solution, usually at a concentration of 3%–5%, was applied to the cervix, and colposcopic images were captured approximately two minutes after application. Low-quality images, including blurry images, images with insufficient brightness, and images with polyps or other structures occluding the cervix, were excluded during quality control. After this quality-control procedure, 5,000 qualified real colposcopic images were retained and used to train FSGAN.

To prevent information leakage and ensure the reliability of subsequent classification model metrics, the dataset used for training the generative adversarial model was strictly segregated from the validation and test sets employed in the classification experiments.

#### 5.2.2 Training process of FSGAN

The training process employs a multi-stage adversarial optimization strategy with meticulously tuned hyperparameters to achieve high-fidelity colposcopic image synthesis. The Adam optimizer was configured with β_1_ = 0.5 and β_2_ = 0.999, utilizing a base learning rate of 2 × 10^−4^ to accommodate the fine-grained generation requirements of cervical lesion features.

The generator and discriminator networks were trained in alternating 1:1 ratio with a batch size of 8. Spatial robustness was enhanced through DiffAugment policy implementation, incorporating color space transformations and translational augmentations to improve model invariance to illumination variations and structural displacements.

**Figure 7:**
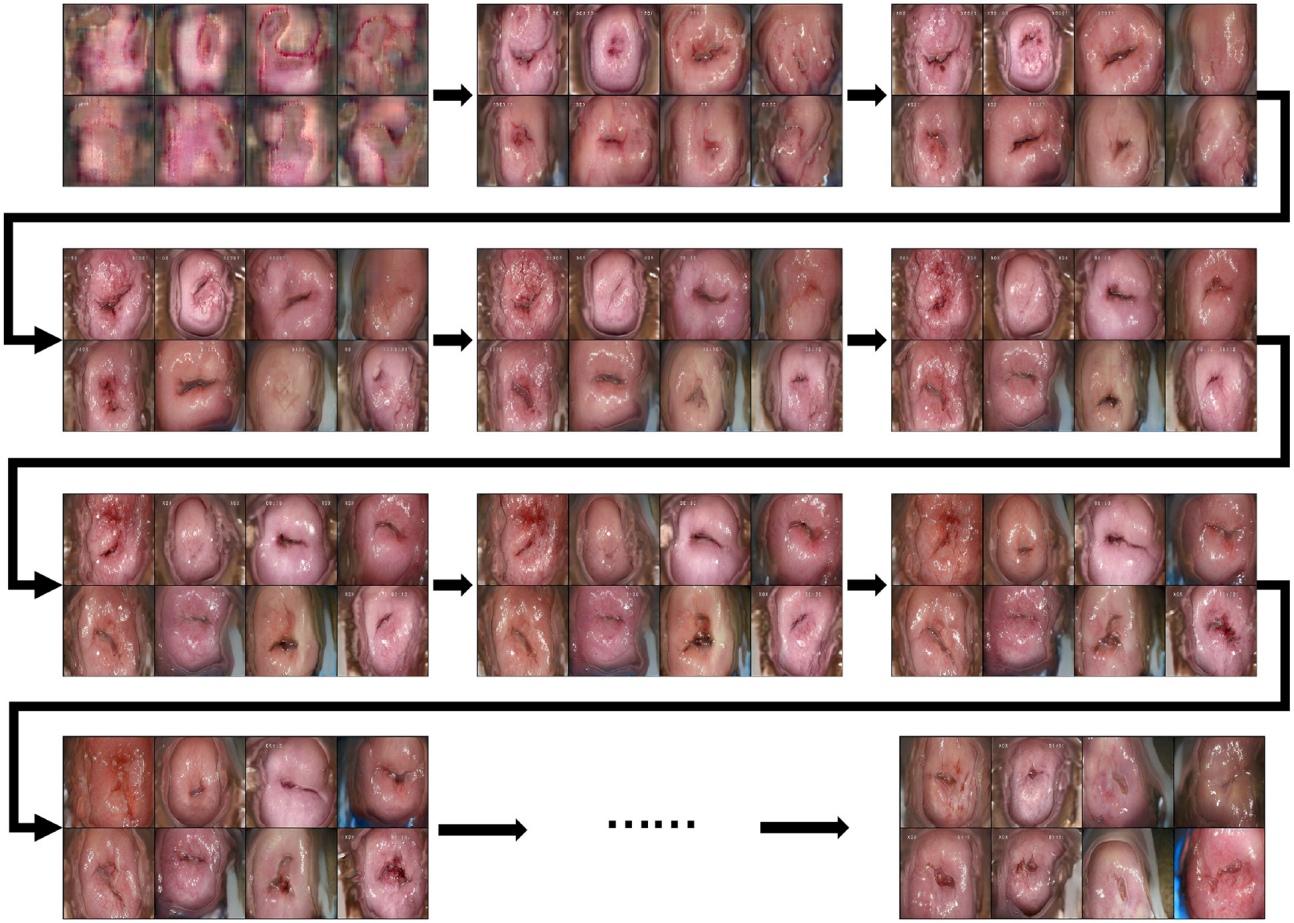
Schematic diagram of FSGAN network training convergence process visualization.

Stability was ensured through progressive training mechanisms: the generator incorporated Exponential Moving Average (EMA) of parameters to mitigate training oscillations, while the discriminator utilized self-supervised reconstruction loss combined with LPIPS (Learned Perceptual Image Patch Similarity) metric to preserve pathological feature semantics. The training convergence progression is visually demonstrated in the following visualization (Figure 7).

#### 5.2.3 FID-based evaluation of FSGAN-generated images

The Fréchet Inception Distance (FID) is adopted as the primary evaluation metric to assess model performance. FID is defined as Equation 7.


$$\begin{array}{l} FID=||\mu_{r}-\mu_{g}{||}_{2}^{2}+\text{Tr}\left(\Sigma_{r}+\Sigma_{g}-2{\left(\Sigma_{r}\Sigma_{g}\right)}^{1/2}\right) \end{array}$$ (7)

where μ_*r*_ and Σ_*r*_ denote the mean and covariance matrix of real-image features, while μ_*g*_ and Σ_*g*_ denote those of generated-image features. A lower FID value indicates that the generated images are closer to real images in terms of feature distribution, image fidelity, and diversity.

FID quantifies generation quality by measuring the distributional discrepancy between real and synthetic images in a deep learning feature space, serving as a core indicator for evaluating generative model performance. Specifically, we employ a pre-trained Inception-v3 network (35) to extract high-dimensional features from both image sets. Lower FID values indicate that the feature distribution of generated images is closer to that of real images, reflecting better image fidelity and diversity.

FID results (Table 1) demonstrate that our proposed FSGAN achieves optimal performance, surpassing both PGGAN and FastGAN in texture detail fidelity. It is worth noting that experimental results show DCGAN and WGAN fail to generate 512*512 resolution images even with repeated stacking of convolutional and activation modules to deepen the network architecture. These two models still suffer from mode collapse during training and fail to converge.

**Table 1:** Comparison of FID values.

Network	FID
PGGAN	38.16
FastGAN	40.83
FSGAN	32.07

As shown in Table 1, FSGAN achieved the lowest FID value of 32.07, outperforming PGGAN and FastGAN, whose FID values were 38.16 and 40.83, respectively. This result indicates that the feature distribution of FSGAN-generated colposcopic images was closer to that of real images. The lower FID score suggests that the proposed feature pyramid connections and cross-layer channel excitation module improved both texture fidelity and distributional consistency. Therefore, the generated images were more suitable for subsequent self-training, providing a more reliable data source for improving the classification performance of CenSwin Transformer. This confirms that FSGAN-generated colposcopic images exhibit the most realistic textural details, providing more reliable data augmentation for subsequent classification models.

### 5.3 Semi-supervised learning and results of CenSwin Transformer

#### 5.3.1 Dataset for CenSwin Transformer

The cervical image dataset was acquired through a multicenter collaboration as stated in Section 5.2. Board-certified gynecologists performed dual annotation, with final dataset inclusion requiring histopathological confirmation through biopsy correlation. The finalized dataset comprised 2,821 low-grade squamous intraepithelial lesions (incorporating normal epithelium and CIN I classifications) and 1,546 high-grade squamous intraepithelial lesions (including CIN II, CIN III, and invasive carcinoma specimens). The data was partitioned through stratified sampling into training (60%), validation (20%), and test (20%) sets while preserving the original class distribution, as detailed in Table 2.

**Table 2:** Dataset.

Category	Low	High	Total
Train	1,694	927	2,621
Validation	564	309	873
Test	564	309	873
Total	2,821	1,546	4,367

#### 5.3.2 Experimental settings

The model was trained with a batch size of 16 using the AdamW optimizer configured with β_1_ = 0.9 and β_2_ = 0.999. The learning rate schedule employed a base rate of 1.25 × 10^−4^ with a 5-epoch warmup period (warmup rate = 1.25 × 10^−7^), followed by cosine annealing decay to a minimum learning rate of 1.25 × 10^−6^. Weight decay was set to 0.1 during warmup, with stochastic depth regularization (drop path rate = 0.2) and gradient clipping (threshold = 1.0) implemented for training stability. Data augmentation included random horizontal flipping with probability 0.5.

The pseudo-labeling phase initiated with a confidence threshold of 0.9, dynamically reduced by 0.05 every 20 epochs (minimum threshold = 0.75). Label refresh cycles were optimized to 10-epoch intervals through empirical validation comparing 5, 10, 20, and 25-epoch intervals. The weight decay coefficient was adjusted to 0.01 during self-training to facilitate finer parameter updates.

#### 5.3.3 Experimental results

##### 5.3.3.1 Comparison experiment between the self-trained CenSwin Transformer and the mainstream classification models

**Figure 8:**
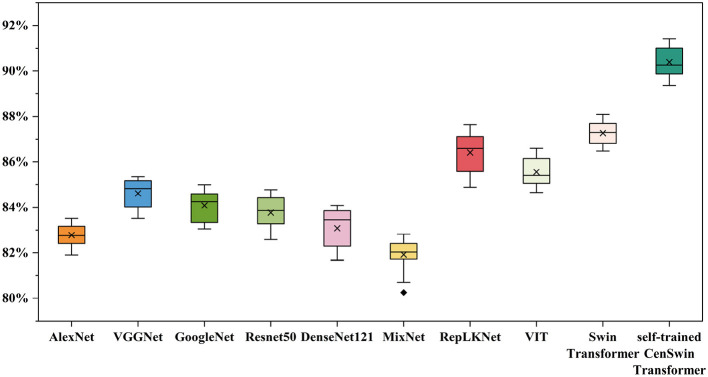
Accuracy box plot over 12 repeated experiments. The comparison was performed on the same stratified test set. CenSwin Transformer showed higher overall accuracy and lower fluctuation than most baseline networks.

**Figure 9:**
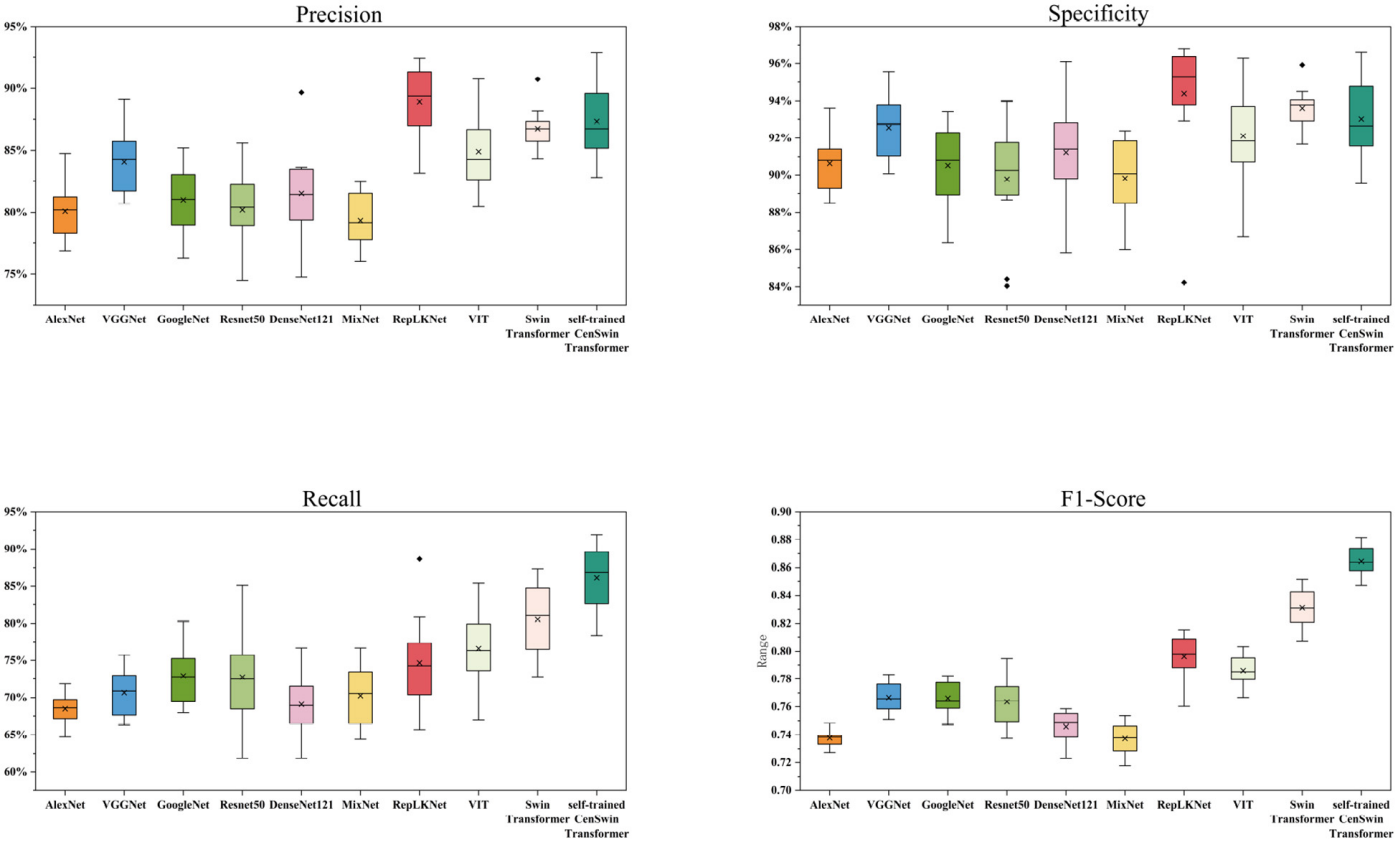
Box plots of precision, specificity, recall, and F1-score over 12 repeated experiments. CenSwin Transformer achieved higher recall and F1-score while maintaining competitive precision and specificity.

The self-trained CenSwin Transformer showed improved classification performance compared with most baseline networks. As shown in Figure 8, the accuracy distribution over 12 repeated experiments indicates that CenSwin Transformer achieved higher overall accuracy and lower fluctuation than most compared models. Box plots of other indicators, including precision, specificity, recall, and F1-score, are shown in Figure 9.

As detailed in Table 3, the proposed CenSwin Transformer achieved the best overall classification performance in terms of accuracy, recall, and F1-score over 12 repeated experiments, while maintaining competitive precision and specificity. Specifically, CenSwin Transformer achieved an accuracy of 90.39 ± 0.67%, recall of 86.10 ± 4.33%, and F1-score of 0.8645 ± 0.0111. Compared with Swin Transformer, which achieved an accuracy of 87.27 ± 0.52% and F1-score of 0.8311 ± 0.0131, the proposed method showed statistically significant improvements in both accuracy and F1-score according to the Wilcoxon signed-rank test. Although RepLKNet achieved the highest precision and specificity, CenSwin Transformer obtained a better balance between recall and F1-score, which is clinically important for reducing missed HSIL cases. The model was also benchmarked against recently published (past 3 years) cervical lesion classification approaches, with quantitative performance metrics detailed in Table 4. Compared with previously reported methods, our method achieved competitive or superior point-estimate performance. Since repeated experimental results and sample-level predictions were not available for the published methods, statistical significance testing was not performed for this literature comparison.

**Table 3:** Comparison with existing classification networks over 12 repeated experiments.

Network	Accuracy	Precision	Recall	Specificity	F1-score	*p*-value Acc.	*p*-value F1
AlexNet	(82.78 ± 0.48)%	(80.11 ± 2.31)%	(68.47 ± 2.03)%	(90.62 ± 1.57)%	0.7378 ± 0.0060	0.0005	0.0005
VGGNet	(84.61 ± 0.66)%	(84.05 ± 2.64)%	(70.66 ± 3.35)%	(92.55 ± 1.75)%	0.7667 ± 0.0107	0.0005	0.0005
GoogleNet	(84.08 ± 0.68)%	(80.99 ± 2.67)%	(72.92 ± 3.82)%	(90.50 ± 2.12)%	0.7662 ± 0.0109	0.0005	0.0005
ResNet50	(83.76 ± 0.70)%	(80.22 ± 3.30)%	(72.76 ± 6.85)%	(89.77 ± 3.02)%	0.7636 ± 0.0183	0.0005	0.0005
DenseNet121	(83.08 ± 0.86)%	(81.51 ± 3.65)%	(69.07 ± 4.20)%	(91.22 ± 2.56)%	0.7459 ± 0.0122	0.0005	0.0005
MixNet (36)	(81.92 ± 0.78)%	(79.31 ± 2.16)%	(70.25 ± 4.14)%	(89.82 ± 2.10)%	0.7374 ± 0.0113	0.0005	0.0005
RepLKNet (37)	(86.42 ± 0.94)%	**(88.93 ± 2.89)%**	(74.67 ± 6.08)%	**(94.39 ± 3.46)%**	0.7961 ± 0.0159	0.0005	0.0005
ViT	(85.55 ± 0.63)%	(84.90 ± 3.18)%	(76.64 ± 5.07)%	(92.11 ± 2.61)%	0.7861 ± 0.0116	0.0005	0.0005
Swin Transformer	(87.27 ± 0.52)%	(86.75 ± 1.68)%	(80.54 ± 4.89)%	(93.62 ± 1.09)%	0.8311 ± 0.0131	0.0005	0.0010
**CenSwin Transformer (Ours)**	**(90.39 ± 0.67)%**	(87.33 ± 3.32)%	**(86.10 ± 4.33)%**	(93.01 ± 2.32)%	**0.8645 ± 0.0111**	–	–

Values are reported as mean ± standard deviation over 12 repeated experiments. The *p*-values were calculated using the Wilcoxon signed-rank test by comparing each method with the proposed CenSwin Transformer. Acc. denotes accuracy. A *p*-value < 0.05 was considered statistically significant. The bolded data represents the maximum value of the data in that column.

**Table 4:** Comparison with other models in the literature.

Method	Acc (%)	Prec (%)	Rec (%)	F1
Improved-Inception based methods resnetv2 (38)	81.24	87.52	81.24	0.8426
GRF-Net (39)	88.49	81.12	85.95	0.8347
RACNet (40)	84.50	84.15	88.35	0.8615
MFEM-CIN (41)	89.20	92.30	88.16	0.9018
EfficientNet-B3 (42)	85.86	85.27	85.18	0.8521
DLRNet (43)	85.72	85.57	85.13	0.8562
Light-SGCNet (44)	83.04	82.77	83.01	0.8333
ShuffleNet_SE (45)	81.38	81.76	80.74	0.8116
Ou (46)	89.90	75.10	80.20	0.7660
Shen and Wang (47)	81.33	79.32	70.94	0.7490
**CenSwin (Ours)**	**90.39**	**87.33**	**86.10**	**0.8645**

The bolded data represents the maximum value of the data in that column.

Overall, the proposed CenSwin Transformer achieved stable and statistically supported performance in cervical precancerous lesion classification. Across 12 repeated experiments, CenSwin Transformer achieved an accuracy of 90.39 ± 0.67%, recall of 86.10 ± 4.33%, and F1-score of 0.8645 ± 0.0111. Compared with Swin Transformer, the improvements in accuracy and F1-score were statistically significant according to the Wilcoxon signed-rank test.

##### 5.3.3.2 Ablation study on FSGAN-assisted self-training

This investigation evaluates the effectiveness of the FSGAN-assisted self-training strategy across three backbone architectures: ResNet50, Swin Transformer, and CenSwin Transformer. The image-generation capability of FSGAN has been independently evaluated using the FID metric in Section 5.2.3. Therefore, this section focuses on whether the FID-validated synthetic images can further improve classification performance when incorporated into the self-training framework. The highest-achieving model iterations were selected for comparative evaluation, revealing consistent performance improvements across all architectures when augmented with self-training, as shown in Table 5.

**Table 5:** Results of self-training ablation experiments over 12 repeated experiments.

Model	Acc.	Prec.	Rec.	Spec.	F1
ResNet50	83.76 ± 0.70	80.22 ± 3.30	72.76 ± 6.85	90.23 ± 3.63	0.7636 ± 0.0183
ResNet50+ST	**85.64 ± 0.90** ^*^	**83.40 ± 4.41**	**77.68 ± 5.70**	**92.34 ± 2.16**	**0.8016 ± 0.0139** ^*^
Swin	87.27 ± 0.52	86.75 ± 1.68	80.54 ± 4.89	93.62 ± 1.09	0.8311 ± 0.0131
Swin+ST	**89.27 ± 0.86** ^*^	**87.60 ± 2.30**	**85.17 ± 4.94**	93.50 ± 2.11	**0.8629 ± 0.0291** ^*^
CenSwin	88.24 ± 0.81	86.32 ± 2.17	80.43 ± 4.35	93.10 ± 1.41	0.8322 ± 0.0273
CenSwin+ST	**90.39 ± 0.67** ^*^	**87.33 ± 3.32**	**86.10 ± 4.33**	93.01 ± 2.32	**0.8645 ± 0.0111** ^*^

Values are reported as mean ± standard deviation over 12 repeated experiments. Acc., Prec., Rec., and Spec. denote accuracy, precision, recall, and specificity, respectively, and are reported in percentage units. ST denotes self-training. The Wilcoxon signed-rank test was used to compare each self-training model with its corresponding baseline model. The superscript ^*^ indicates statistically significant improvement in accuracy and F1-score. A *p*-value < 0.05 was considered statistically significant. The bolded data represents the maximum value of the data in that column.

As shown in Table 5, the FSGAN-assisted self-training strategy consistently improved the overall classification performance of all three backbone networks. For ResNet50, self-training increased the accuracy from 83.76 ± 0.70% to 85.64 ± 0.90%, and the F1-score from 0.7636 ± 0.0183 to 0.8016 ± 0.0139. For Swin Transformer, self-training improved the accuracy from 87.27 ± 0.52% to 89.27 ± 0.86%, and the F1-score from 0.8311 ± 0.0131 to 0.8629 ± 0.0291. For CenSwin Transformer, self-training further improved the accuracy from 88.24 ± 0.81% to 90.39 ± 0.67%, and the F1-score from 0.8322 ± 0.0273 to 0.8645 ± 0.0111. The Wilcoxon signed-rank test showed that the improvements in accuracy and F1-score were statistically significant for all three models. Although precision and specificity showed slight fluctuations in some cases, self-training substantially improved recall and F1-score, suggesting that it enhanced the model's sensitivity to positive samples and improved the balance between precision and recall.

**Figure 10:**
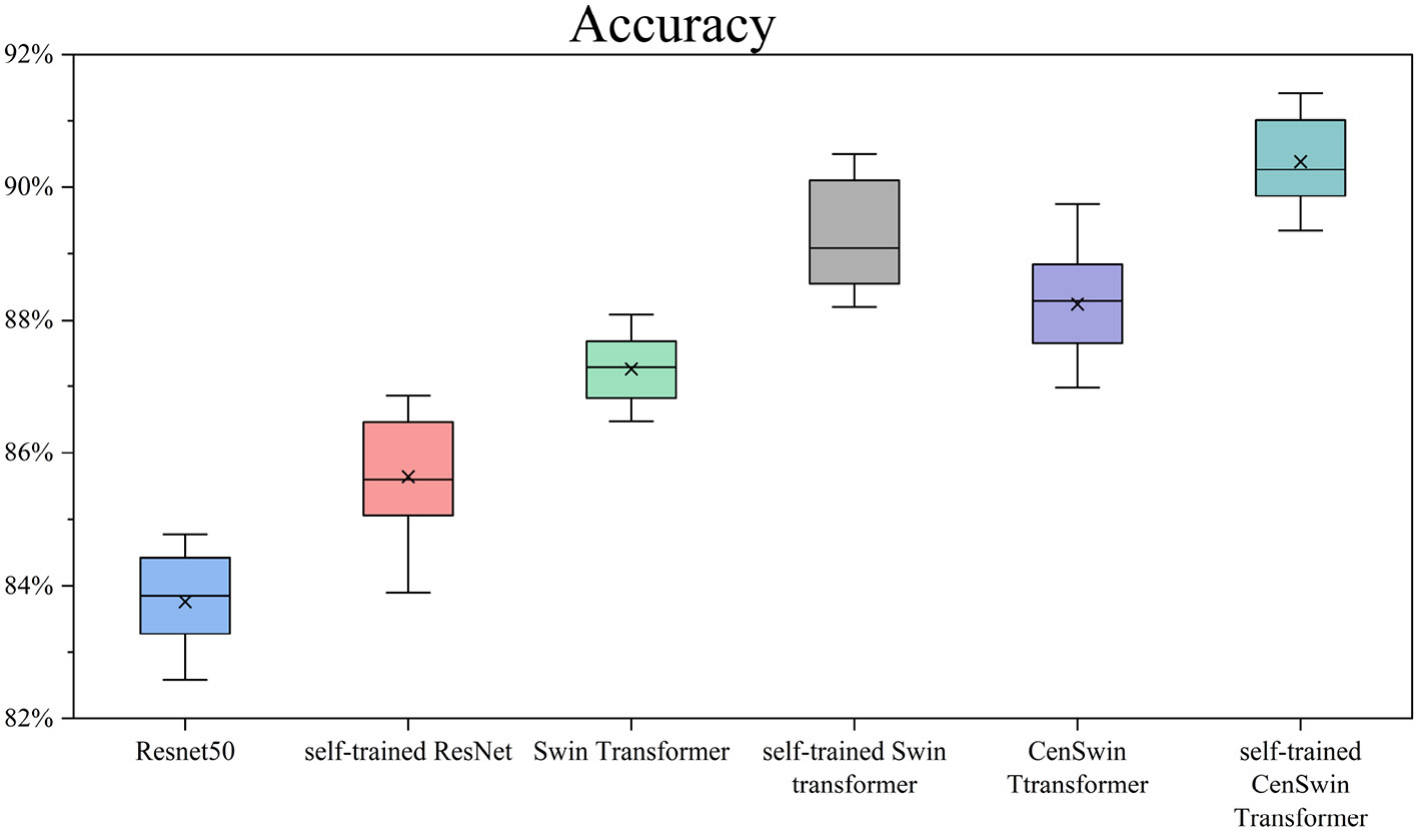
Accuracy box plot before and after self-training over 12 repeated experiments. Self-training improved the accuracy distributions of ResNet50, Swin Transformer, and CenSwin Transformer, indicating that the FSGAN-assisted self-training strategy enhanced classification performance across different backbone networks.

**Figure 11:**
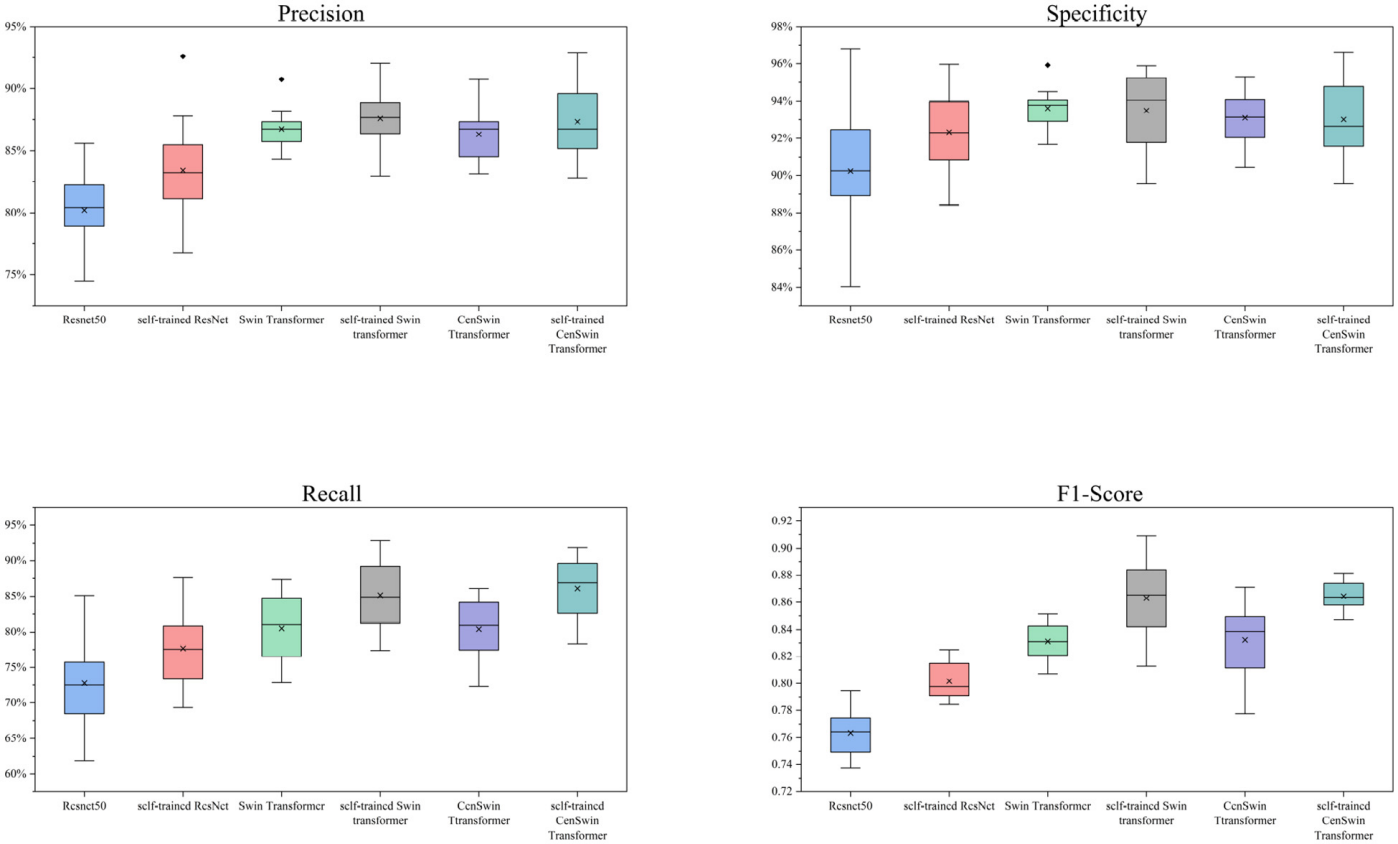
Precision, recall, specificity, and F1-score box plots before and after self-training. Self-training improved recall and F1-score across the evaluated backbone networks and reduced performance fluctuation in repeated experiments.

Figure 10 compares the accuracy distributions of different models before and after self-training, and Figure 11 presents the corresponding distributions of precision, recall, specificity, and F1-score. These results further show that self-training improved recall and F1-score across the evaluated backbone networks and reduced performance fluctuation in repeated experiments.

##### 5.3.3.3 Effect of the generated images from FSGAN

**Figure 12:**
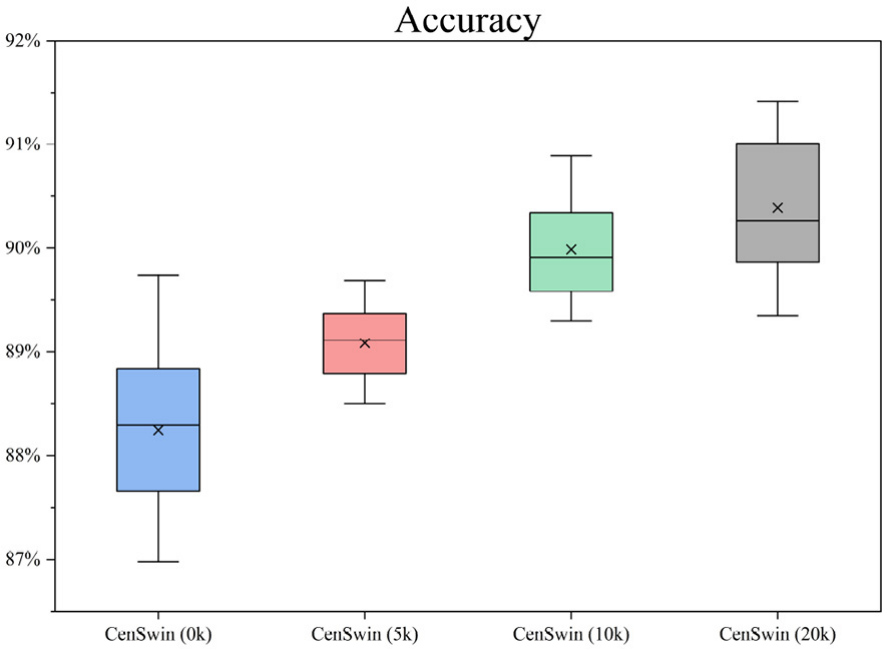
Accuracy box plot. The mean accuracy increased from 88.24% in the baseline setting to 89.09%, 89.99%, and 90.39% when 5K, 10K, and 20K generated images were used, respectively. The results indicate that generated images improved the overall classification accuracy, although the marginal gain gradually decreased as the number of generated images increased.

**Figure 13:**
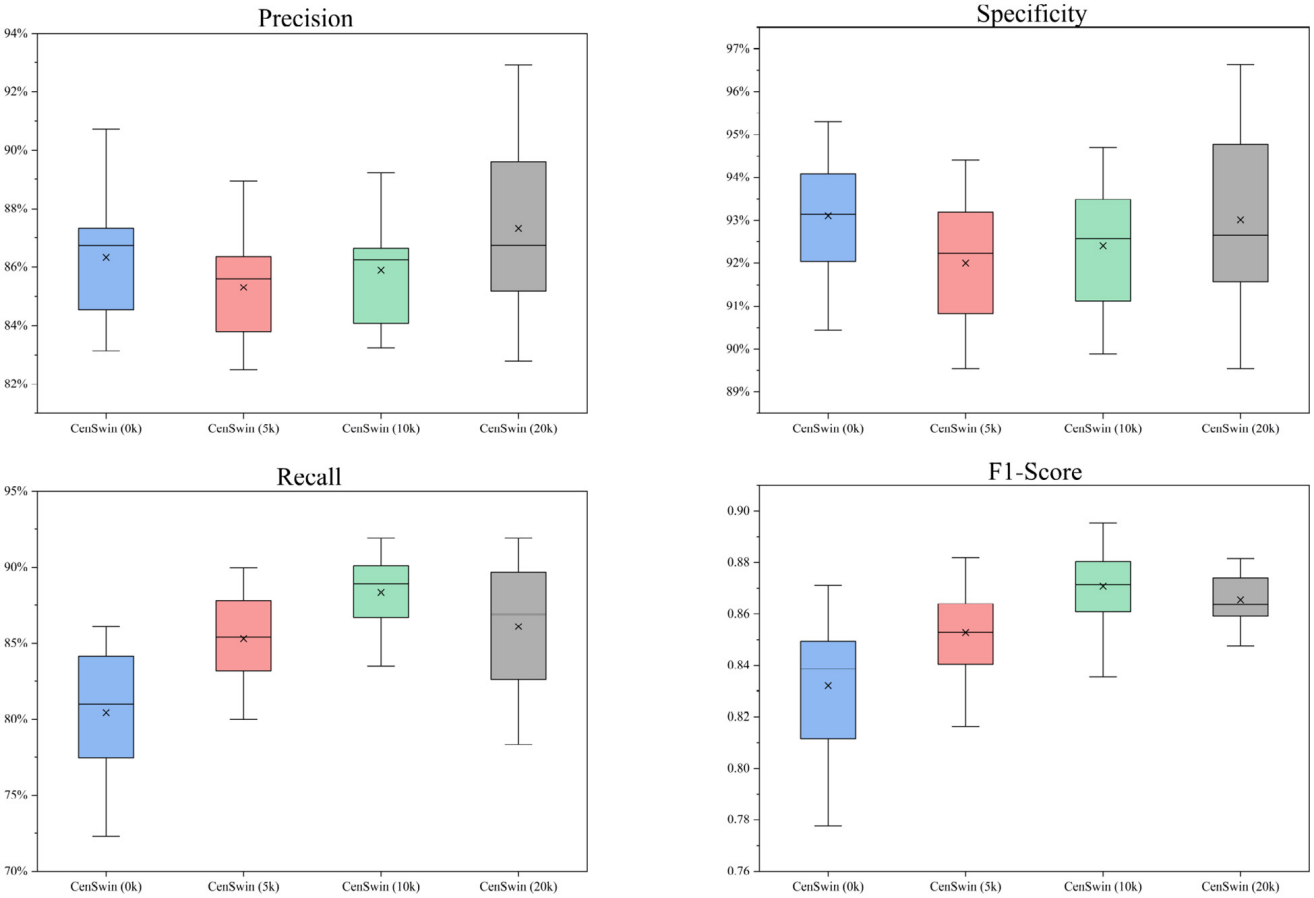
Precision, Recall, Specificity, and F1-Score box plots. The F1-score improved from 0.8322 in the baseline model to 0.8527, 0.8708, and 0.8656 with 5K, 10K, and 20K generated images, respectively. Recall reached its highest value with 10K generated images and slightly decreased with 20K generated images, suggesting that excessive synthetic samples may introduce distributional shift and do not necessarily improve every metric monotonically.

Figures 12 and 13 show the effect of different amounts of FSGAN-generated data on the performance of CenSwin Transformer during self-training. As shown in Table 6, incorporating generated images significantly improved the classification performance of CenSwin Transformer. Compared with the baseline model, the use of 5K, 10K, and 20K generated images consistently improved accuracy and F1-score, and all improvements were statistically significant according to the Wilcoxon signed-rank test. Specifically, the accuracy increased from 88.24 ± 0.81% to 89.09 ± 0.37%, 89.99 ± 0.50%, and 90.39 ± 0.67% when 5K, 10K, and 20K generated images were used, respectively. Meanwhile, the F1-score increased from 0.8322 ± 0.0273 to 0.8527 ± 0.0190, 0.8708 ± 0.0168, and 0.8656 ± 0.0098, respectively. Notably, 20K generated images achieved the highest accuracy and precision, whereas 10K generated images achieved the highest recall and F1-score. The recall increased from 80.43 ± 4.35% in the baseline model to 88.35 ± 2.66% with 10K generated images, but slightly decreased to 86.10 ± 4.33% when 20K generated images were used. This suggests that increasing the number of generated images improves overall performance but does not necessarily lead to monotonic improvement in every metric. Although FSGAN-generated images improved the overall performance of the classifier, the number of generated images used for self-training was larger than the number of real labeled training images. This may introduce a potential distributional imbalance if synthetic samples are overrepresented during training. In particular, excessive generated images may cause the model to learn synthetic-specific texture patterns that do not fully match real clinical images, thereby affecting calibration and generalization to genuinely unseen data. Therefore, the proportion of generated images should be carefully controlled in future self-training strategies.

**Table 6:** Effect of different numbers of FSGAN-generated images.

Setting	Acc.	Prec.	Rec.	Spec.	F1
CenSwin baseline	88.24 ± 0.81	86.32 ± 2.17	80.43 ± 4.35	**93.10 ± 1.41**	0.8322 ± 0.0273
+ 5K images	**89.09 ± 0.37** ^*^	85.31 ± 1.87	85.30 ± 3.10	92.00 ± 1.61	**0.8527 ± 0.0190** ^*^
+ 10K images	**89.99 ± 0.50** ^*^	85.90 ± 1.91	**88.35 ± 2.66**	92.41 ± 1.54	**0.8708 ± 0.0168** ^*^
+ 20K images	**90.39 ± 0.67** ^*^	**87.33 ± 3.32**	86.10 ± 4.33	93.01 ± 2.32	0.8656 ± 0.0098^*^

Values are reported as mean ± standard deviation over 12 repeated experiments. Acc., Prec., Rec., and Spec. denote accuracy, precision, recall, and specificity, respectively, and are reported in percentage units. The Wilcoxon signed-rank test was used to compare each generated-image setting with the CenSwin baseline. The superscript ^*^ indicates statistically significant improvement in accuracy and F1-score compared with the baseline. For 5K images, *p* = 0.0010 for accuracy and *p* = 0.0005 for F1-score; for 10K images, *p* = 0.0005 for both accuracy and F1-score; for 20K images, *p* = 0.0005 for both accuracy and F1-score. A *p*-value < 0.05 was considered statistically significant. The bolded data represents the maximum value of the data in that column.

**Figure 14:**
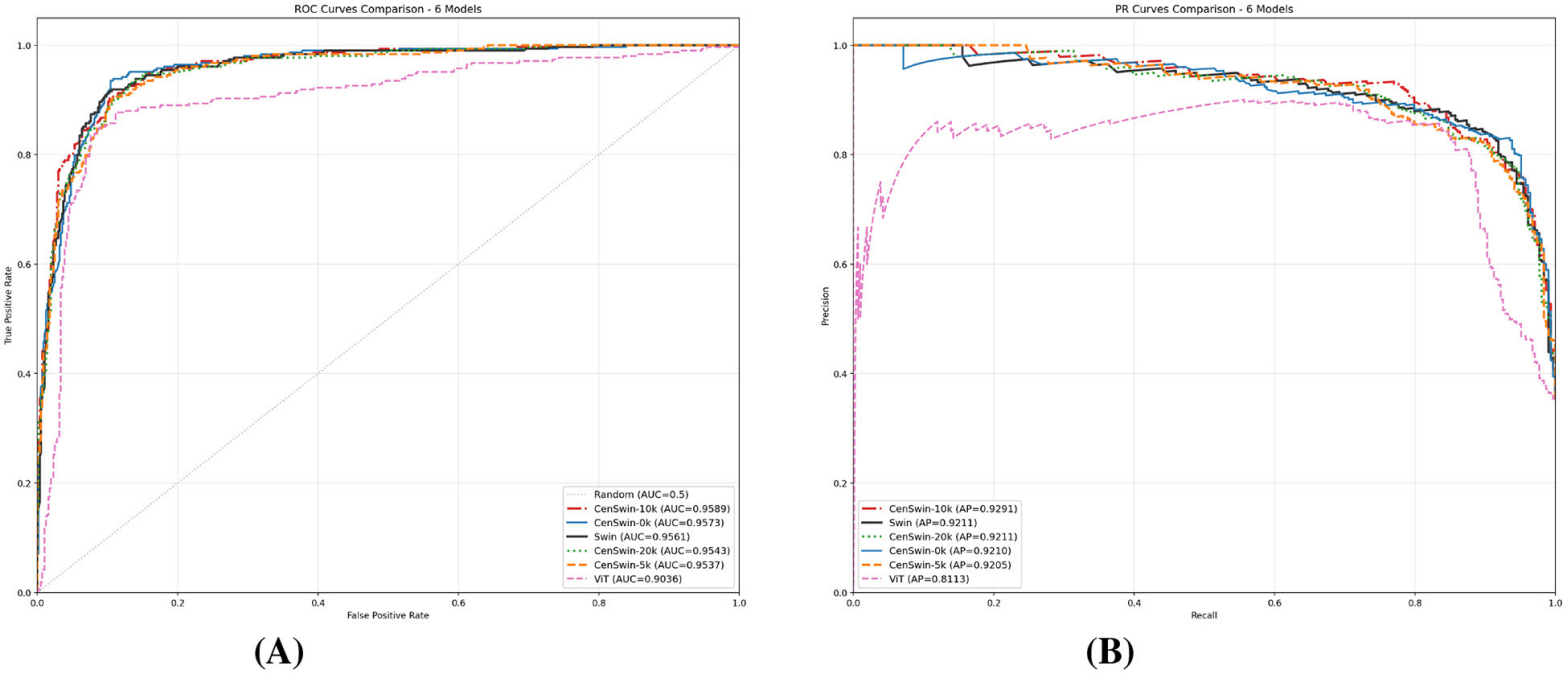
ROC and PR curves of representative models and generated-image settings. **(A)** ROC curves of ViT, Swin Transformer, CenSwin-0K, CenSwin-5K, CenSwin-10K, and CenSwin-20K. **(B)** PR curves of the same settings. ROC-AUC and PR-AUC were used to evaluate threshold-independent discriminative performance. CenSwin-10K achieved the highest ROC-AUC and PR-AUC values among the evaluated settings. **(A)** ROC curves. **(B)** PR curves.

To further evaluate threshold-independent discriminative performance, ROC and PR curves were plotted for representative models and generated-image settings, including ViT, Swin Transformer, CenSwin-0K, CenSwin-5K, CenSwin-10K, and CenSwin-20K. As shown in Figure 14, CenSwin-10K achieved the highest ROC-AUC and PR-AUC values among the evaluated settings, reaching 0.9589 and 0.9291, respectively. CenSwin-0K also showed strong discriminative performance, with a ROC-AUC of 0.9573 and a PR-AUC of 0.9210, while Swin Transformer achieved a ROC-AUC of 0.9561 and a PR-AUC of 0.9211. In contrast, ViT showed lower threshold-independent performance, with a ROC-AUC of 0.9036 and a PR-AUC of 0.8113.

These results further support the discriminative ability of the proposed CenSwin architecture. Meanwhile, the generated-image settings showed a non-monotonic trend: CenSwin-10K achieved the best ROC-AUC and PR-AUC, whereas CenSwin-5K and CenSwin-20K showed values comparable to the CenSwin-0K baseline. This finding is consistent with the results in Table 6, suggesting that FSGAN-generated images can provide useful auxiliary information for self-training when used at an appropriate scale, but increasing the number of generated images does not necessarily lead to continuous improvement. Since ROC-AUC and PR-AUC were calculated for representative models and core generated-image settings rather than all baseline networks, these results should be interpreted as a supplementary threshold-independent analysis.

##### 5.3.3.4 Comparison with the Doctor's diagnosis under the independent test set

To further evaluate the auxiliary diagnostic value of the proposed model, we compared its performance with that of six gynecologists on an independent dataset of 380 static colposcopic images. None of these images participated in model training or validation. Biopsy results were used as the reference standard, and both the gynecologists and the model evaluated the same images. It should be noted that this experiment was designed as an image-only comparison rather than a complete clinical colposcopy assessment. The gynecologists evaluated static images without access to complete clinical information such as HPV results, cytology, patient history, or dynamic live colposcopy findings.

As shown in Table 7, the proposed model achieved higher accuracy, recall, precision, and F1-score than the six gynecologists under the image-only evaluation setting. Notably, the model obtained a recall of 71.11%, whereas the doctors' recall ranged from 17.04% to 40.74%, indicating that the model was more sensitive in identifying high-risk cervical lesions and may help reduce missed HSIL cases.

**Table 7:** Doctor-model comparison experiment on an independent dataset.

Evaluator	Accuracy	Precision	Recall	Specificity	F1-Score
Doctor A	67.37%	56.96%	33.33%	86.12%	0.4206
Doctor B	65.53%	52.50%	31.11%	84.49%	0.3907
Doctor C	57.58%	73.47%	38.71%	81.94%	0.5070
Doctor D	68.68%	59.76%	36.30%	86.53%	0.4516
Doctor E	68.16%	57.29%	40.74%	83.27%	0.4762
Doctor F	67.89%	69.70%	17.04%	95.92%	0.2738
**Our Model**	**85.26%**	**84.96%**	**71.11%**	**93.06%**	**0.7742**

The independent dataset contained 380 static colposcopic images that were not used during model training or validation. Biopsy results were used as the reference standard. Recall/sensitivity reflects the ability to identify HSIL-positive cases, while specificity reflects the ability to identify LSIL/negative cases. F1-score reflects the balance between precision and recall. The bolded data represents the maximum value of the data in that column.

However, the recall of the model on this independent dataset was lower than that on the main test set. This decrease may be related to domain shift caused by differences in image acquisition devices, illumination conditions, patient populations, lesion distribution, and hospital-specific imaging protocols. In addition, both the model and doctors evaluated static images without complete clinical information or dynamic colposcopic examination, which may further increase diagnostic difficulty. Therefore, the model should be regarded as an auxiliary second-opinion tool for colposcopic image interpretation rather than a replacement for clinicians.

## 6 Limitations

This study has several limitations. First, the classification results were based on a single stratified train-validation-test split. Although 12 repeated experiments were performed to evaluate training stability under different random initializations, the test-set composition did not change across runs. Future studies will include k-fold cross-validation and additional external multicenter validation to provide a more robust estimate of model generalization.

Second, ROC-AUC and PR-AUC analyses were added for representative models and core generated-image settings, including ViT, Swin Transformer, CenSwin-0K, and CenSwin with 5K, 10K, and 20K generated images. However, ROC-AUC and PR-AUC comparisons were not exhaustively performed for all competing baseline networks. Future studies will retain complete sample-level prediction probabilities for all models to enable comprehensive ROC-AUC, PR-AUC, and DeLong test analyses.

Third, the current ablation study mainly evaluates the combined effect of FSGAN-generated images and pseudo-label-based self-training. The independent contributions of generated images and the self-training procedure were not fully separated. Future work will include more fine-grained ablation settings, such as supervised training with generated images but without pseudo-label updating, and self-training without generated images, to further disentangle the effects of each component.

Finally, the comparison with gynecologists was conducted under an image-only setting using static colposcopic images. Complete clinical information and dynamic colposcopy findings were not available during this comparison. Therefore, the results should be interpreted as evidence of potential auxiliary value rather than a direct replacement for full clinical colposcopic assessment.

## 7 Conclusion

FSGAN, a generative adversarial network is proposed in this paper, combining feature pyramid network and cross-layer channel excitation for multi-scale feature fusion. Realistic high-resolution images similar to the cervical structure under real colposcopy can be generated using FSGAN to effectively expand the training data.

A semi-supervised collaborative training framework is adopted for CenSwin transformer. CenSwin Transformer, a lesion classification network that mimics the mechanism of human vision to suppress the interference of edge noise in colposcopic images and enhance the feature extraction of cervical transformation area, is proposed.

Across 12 repeated experiments, the proposed CenSwin Transformer with self-training achieved an accuracy of 90.39 ± 0.67%, recall of 86.10 ± 4.33%, and F1-score of 0.8645 ± 0.0111, demonstrating improved and statistically supported classification performance compared with baseline models. These results indicate that the combination of GCPE, convolutional patch embedding, and FSGAN-assisted self-training can enhance lesion-related feature representation in colposcopic images.

On the independent dataset of 380 static colposcopic images, the model achieved 85.26% accuracy, 71.11% recall, and an F1-score of 0.7742. Compared with the gynecologists under the image-only evaluation setting, the model showed higher recall and F1-score, suggesting its potential as an auxiliary second-opinion tool for colposcopic image interpretation. However, the model is not intended to replace clinicians, and further multicenter validation is still needed before clinical deployment.

## Data Availability

The raw data supporting the conclusions of this article will be made available by the authors, without undue reservation.
